# Correlation of Residual Symptoms With Triiodothyronine (T3) in Patients Treated for Hypothyroidism

**DOI:** 10.7759/cureus.78095

**Published:** 2025-01-27

**Authors:** Altaf Ali Naushad, Chirag LU, Nikitha S, Sagar Sourabh, Bharathi Kolla, Chitra Selvan, Pramila Kalra, Manjunath PR, Ganavi YP

**Affiliations:** 1 Endocrinology, Meitra Hospital, Calicut, IND; 2 Endocrinology, Diabetes and Metabolism, Kempegowda Institute of Medical Sciences (KIMS) Hospital and Research Centre, Bengaluru, IND; 3 Endocrinology, M.S. Ramaiah Medical College, Bengaluru, IND; 4 Endocrinology and Metabolism, M.S. Ramaiah Medical College, Bengaluru, IND

**Keywords:** ghq-12, hads-a, levothyroxine, lt4, persistent symptoms, residual, t4, triiodothyronine (t3), tsq

## Abstract

Background

Despite the time-honored concept that a normal serum thyroid-stimulating hormone (TSH) indicates that there is euthyroidism in all tissues, there is accumulating evidence questioning this notion. This could explain why a subset of patients remain symptomatic despite treatment with levothyroxine. This study will help identify patients with residual symptoms through questionnaires and the relation of the test scores (obtained from these questionnaires) to serum triiodothyronine (T3) levels.

Methods

A total of 108 hypothyroid patients on levothyroxine supplementation who are biochemically euthyroid were included. Residual symptoms on treatment and symptom questionnaires (thyroid symptom questionnaire (TSQ), general health questionnaire (GHQ-12), Hospital Anxiety and Depression Scale (HADS) and Epworth Sleepiness Score (ESS)) were administered to subjects and their scores correlated with T3 values.

Results

A total of 108 subjects, comprising 98 females (90.7%) and 10 males (9.3%), with a mean age of 39.19 years (± 13.18 years), were analyzed in this study. The majority of the participants (75.9%, n=82) reported experiencing at least one symptom. The most prevalent symptom was dry skin, reported by 44.4% (n=48) of the subjects, followed by weight gain, which affected 30.6% (n=33) of the participants. Statistical analysis revealed significant inverse correlations between all assessed scores - TSQ, GHQ-12, HADS, and ESS - with T3 values. The correlation coefficients and corresponding p-values indicate significant relationships between thyroid function and psychological well-being in the studied population. The following correlation coefficients (r values) were obtained: TSQ, r=-0.331 (p = 0.001); GHQ-12, r=-0.292 (p = 0.002); HADS-A, r=-0.318 (p = 0.001); HADS-D, r = -0.233 (p = 0.015); and the ESS, r=-0.205 (p = 0.034). These findings highlight the notable impact of thyroid function on mental health outcomes.

Conclusions

Treatment satisfaction remains suboptimal for patients with residual symptoms of hypothyroidism. TSQ especially seems to be a useful tool to recognize these residual symptoms. The further use of this tool to identify patients with residual symptoms may enable future studies to test the role of oral T3 in such patients given the strong negative correlation.

## Introduction

Hypothyroidism affects between 5% and 10% of the population in India with upwards of 40 million people in the country under treatment with levothyroxine (LT4) supplementation [1,2]. Ever since the use of LT4 as the standard of treatment for hypothyroidism in the 1970s, it has been well documented that a subset of patients under treatment with LT4 have residual symptoms even after normalization of serum thyroid-stimulating hormone (TSH), the standard treatment target metric [3]. Thus, treatment satisfaction remains suboptimal despite the condition being “easy-to-detect and inexpensive to treat.” Globally, the prevalence of patients having residual symptoms to treatment with LT4 is estimated to be between 10% and 20% [4], with wide variation based on the symptoms.

The persistence of residual symptoms has called into question the utility of management of hypothyroidism with levothyroxine alone, even though levothyroxine itself may not be achieving euthyroidism in all tissues. This is explained partly by the fact that only 20% of the circulating triiodothyronine (T3) is produced by the thyroid gland (with the rest by peripheral conversion of thyroxine (T4)) and by tissue-specific differences in the conversion of T4 to T3. The latter is largely due to polymorphisms in deiodinase enzymes and heterogeneity of patients with respect to their residual thyroid function and individual setpoints for optimal thyroid homeostasis [5]. Indeed, in the past, thyroid extracts containing a combination of T4 and T3 were widely used and are still available in various parts of the world; however, their popularity has declined due to several factors, not least of which were side effects due to inconsistency in levels of T3 [6]. 

With the recent widespread availability of stand-alone oral T3 with precise dosing, the possibility of its combination with LT4 in selected patients may be preferred since T3 supplementation may theoretically be useful in the amelioration of residual symptoms in such populations. Though the evidence showing superiority of T4+T3 is lacking, interest in the use of this combination continues to grow especially in the context of residual or persistent symptoms after normalization of TSH. A 2006 meta-analysis of 11 randomized clinical trials comprising 1216 patients showed no benefit of T4+T3 over monotherapy with T4 with no significant difference regarding safety or effectiveness [7]. On the other hand, patients do report a better quality of life when on the combination therapy [8]. The Joint British Thyroid Association/Society for Endocrinology recently suggested the use of T3 in non-pregnant adults after a careful selection of patients having residual symptoms without other comorbidities [9].

In order to address the problem of treating patients with residual symptoms of hypothyroidism, it is essential to first objectively understand its prevalence and severity. In addition, a means to carefully select those with residual symptoms among whom T3 supplementation would be a beneficial tool. This study was performed to determine the prevalence and severity of the residual symptoms and to understand the correlation, if any, of T3 levels in LT4-treated hypothyroid patients who are biochemically euthyroid with various symptom questionnaire scores. The symptom questionnaires looked at several domains pertaining to hypothyroid symptomatology in order to address a wide range of residual symptoms.

## Materials and methods

This study was a cross-sectional questionnaire-based study that was conducted in the endocrinology department outpatient clinic of a tertiary care centre in Bengaluru, India. The recruitment of patients was done between November 2022 and August 2023. All consecutive cases of hypothyroidism who are biochemically euthyroid and on a stable dose of LT4 for the past six months were recruited. After informed consent, subjects underwent a brief history elicitation, physical examination, and measurement of anthropometric values. Patient information on seven residual symptoms commonly attributed to hypothyroidism, each with a brief description provided for consistency, was collected. These, chosen from previous studies, included diminished sweating, hoarseness, paresthesias, dry skin, constipation, impaired hearing and increase in weight. They were then subjected to four self-administered questionnaires detailed below. Data regarding current TSH, thyroxine (T4) and triiodothyronine (T3) levels were collected for analysis.

Cases

For this study, 108 consecutive patients who were on LT4 supplementation for primary hypothyroidism within the age range of 18-75 years were recruited after obtaining their informed consent. The subjects included were biochemically euthyroid as per the American Thyroid Association (ATA) guidelines (TSH: 0.4-4 mIU/L) for at least the past six months and on a stable LT4 dose. Excluded were pregnant patients, those with recent hospitalization in the last six months, those with central hypothyroidism, post-radioiodine ablation and thyroidectomized patients. Subjects with major comorbidities including coronary artery disease, type 2 diabetes, chronic obstructive pulmonary disease, malabsorption disorders, gastrointestinal surgeries, significant renal or liver dysfunction, seizure disorders, any active cancer, history of psychiatric disease, and on psychotropic medications were also excluded. Also, patients who were on medication known to affect thyroid hormone metabolism and/or bioavailability in any way including corticosteroids, amiodarone, iron supplements, sucralfate, proton pump inhibitors, cholestyramine, oral contraceptive pills (OCPs) or any co-prescription of treatment were excluded as well. 

Questionnaires

A battery of four validated questionnaires was used to assess major domains pertaining to the symptomatology of hypothyroidism including the thyroid symptom questionnaire (TSQ) [10], general health questionnaire (GHQ-12) [11], Hospital Anxiety and Depression Scale (HADS-A and HADS-D) [12], and the Epworth Sleepiness Score (ESS) [13]. The TSQ is a compendium of common symptoms related to hypothyroidism derived from the symptoms reported by patients to the British Thyroid Foundation Newsletter designed to elicit how patients feel on LT4 replacement [14]. Higher scores on TSQ reflect poorer treatment satisfaction. GHQ-12 is a 12-point questionnaire with abridged versions, including the 60-, 30-, 28-, and 12-item versions; it is a general screening for the assessment of mental health status and psychiatric morbidity [15]. The 12-point version is short and easy to self-administer and is the most widely used format and scoring was done along a four-point ordinal scale, with higher scores reflecting poorer mental health status. HADS is a compound self-assessment tool for both anxiety and depression [16], two domains which are well known to be affected by hypothyroidism. Scoring for both the above questionnaires was on a Likert scale from 0 to 3, with higher scores reflecting possible anxiety and depression. As noted by Kalra et al. [17], clinical scoring scales play a crucial role in evaluating various aspects of thyroid dysfunction, including psychological well-being, thereby highlighting the importance of these assessments in comprehensive thyroidology practice. ESS is a brief questionnaire used to assess daytime sleepiness, particularly in the context of obstructive sleep apnea (OSA). It consists of eight items where respondents rate their likelihood of dozing off in various everyday situations on a scale from 0 (would never doze) to 3 (high chance of dozing), resulting in a total score ranging from 0 to 24. Scores below 10 indicate normal daytime sleepiness, while scores of 10 or higher suggest excessive sleepiness that may necessitate further evaluation for OSA.

Hormonal assessment

Biochemical testing for all the subjects (TSH, T4 and T3) was done and analyzed in the Central Laboratory of M.S. Ramiah Medical College. Serum TSH analysis was done by electrochemiluminescence immunoassay (ECLIA) method (VITROS ECi/ECiQ/3600, third generation, Cardinal Health, Dublin, OH, USA) with the limit of detection (LOD) of 0.01 μIU/mL. The inter- and intra-assay variability was 2.9 and 3. T4 and T3 were tested by ECLIA (VITROS ECi/ECiQ 3600) with a limit of detection (LOD), respectively, of 5.21 nmol/L (0.405 mcg/dL) and 0.196 nmol/L (0.128 ng/mL).

Ethics

The study was approved by the Institutional Review Board (IRB) of the M.S. Ramaiah Medical College (Reg no. ECR/215/Inst/KA/2013/RR-19) vide letter no MSRMC/EC/SP-04/10-2022 on October 27, 2022. Written informed consent from the subjects was obtained for participation in the study and for the use of the patient data for research and educational purposes.

Statistical analysis

We employed descriptive statistics to summarize demographic and clinical data, presenting continuous variables as mean±standard deviation (SD) and categorical variables as frequencies and percentages. For assessing the correlations between T3 values and questionnaire scores, Spearman’s correlation was utilized due to its suitability for nonparametric data.

Singh et al. observed that the symptom complex among hypothyroid patients was 57.6% for ‘lack of energy’ [18]. In the present study, expecting a similar result, with a 95% confidence level and absolute precision of 8%, the present study requires a minimum of 108 subjects. Descriptive statistics of demographic and clinical data were calculated to identify the trends and correlations among various symptoms experienced by the participants. The significance of the correlation of symptoms with the questionnaire was exploratory in this study. All correlation analyses were two-tailed. For comparisons of the means between two related groups, Student’s t-test was utilized. P<0.05 was considered statistically significant. All data were analyzed using SPSS, version 16 (SPSS Inc., Chicago, IL, USA).

## Results

Baseline characteristics and questionnaire scores

The study cohort consisted of 108 subjects, including 98 women (90.7%) and 10 men (9.3%), with a mean age of 39.19 years (± 13.18 years; age range: 18 to 71 years). The mean values for TSH, T4, and T3 were as follows: TSH: 2.38 mIU/mL (±1.15 mIU/mL), T4: 9.6 mcg/dL (±1.89 mcg/dL), and T3: 0.97 ng/mL (±0.21 ng/mL). The baseline characteristics, expressed as mean±standard deviation (SD), are presented in Table 1.

**Table 1:** Baseline characteristics of study cohort and mean scores on questionnaires TSH: thyroid-stimulating hormone; T4, thyroxine; T3, triiodothyronine; GHQ, General Health Questionnaire; TSQ, thyroid symptom questionnaire; HADS, Hospital Anxiety and Depression Scale; ESS, Epworth Sleepiness Score.

Variable (n=108)	Mean±SD	Range
Age (years)	39.19±13.18	18-71
BMI (kg/m^2^)	26.83±4.93	16.9-40.9
TSH (mIU/mL)	2.38±1.15	0.4-4.0
T4 (mcg/dL)	9.6±1.89	5.38-17.61
T3 (ng/mL)	0.97±0.21	0.5-1.64
TSQ (max. score: 36)	9.99±6.48	0-28
GHQ-12 (max. score: 36)	9.83±5.15	0-33
HADS-A (max. score: 21)	4.39±4.02	0-16
HADS-D (max. score: 21)	3.09±3.03	0-12
ESS (max. score: 24)	3.76±2.66	0-12

The mean score on the TSQ was 9.99±6.48 (range: 0-28 on a maximum score of 36). The mean GHQ-12 score was 9.83±5.15 (range: 0-33 with a maximum score of 36). The score on HADS-A and HADS-D assessment for anxiety and depression respectively was 4.39±4.02 and 3.09±3.03 (range: 0-16 and 0-12 respectively, maximum score of 21 each). For an assessment for obstructive sleep apnea by ESS, the mean score was 3.76±2.66 (range:0-12, maximum score of 24).

Residual symptoms

**Figure 1:**
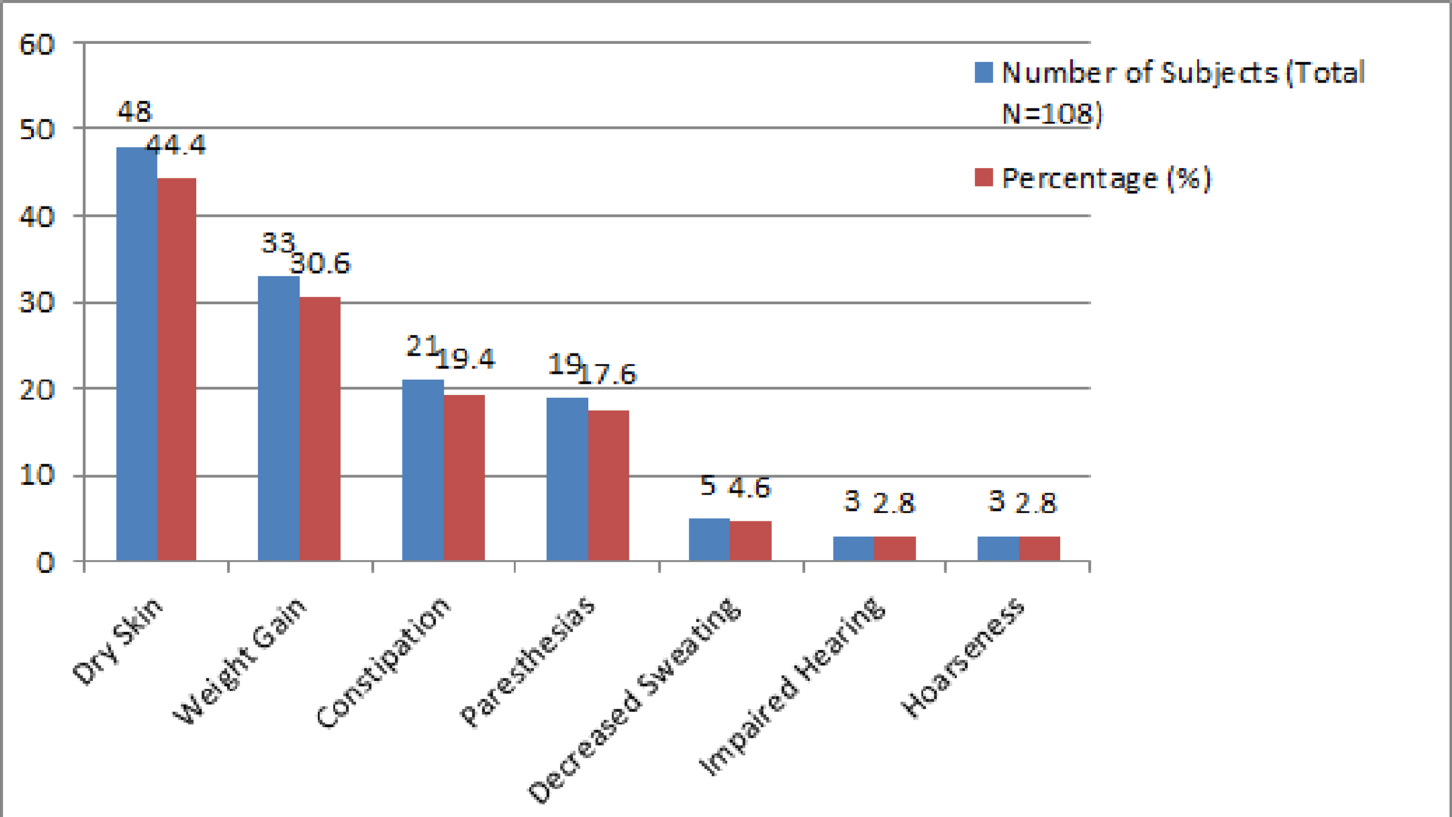
Symptom frequency among biochemically euthyroid subjects (N= 108) Graph created by Dr. Altaf Ali Naushad.

Among the 108 subjects, the residual symptoms analyzed included the following: decreased sweating, hoarseness, dry skin, paresthesias, constipation, impaired hearing, and weight gain. The most common symptom reported was dry skin by 48 participants (44.4%) followed by weight gain in 33 subjects (30.6%), constipation in 21 (19.4%), paresthesias in 19 (17.6%), decreased sweating in five (4.6%), impaired hearing in three (2.8%), and hoarseness in three participants (2.8%) (Figure 1).

Comparison of T3 by residual symptoms

Using the Student's t-test, comparing the presence or absence of individual symptoms with T3 values, none of the symptoms showed statistical significance except for impaired hearing (P= 0.044), which was reported in 2.8% of the subjects (Table 2).

**Table 2:** Comparison of the presence (yes) or absence (no) of symptoms with mean serum T3 (ng/ml) levels of patients P-value calculated by Student's t-test. T3, triiodothyronine.

Symptom	Yes		No		P Value
	Mean T3 (ng/ml)	SD	Mean T3 (ng/ml)	SD	
Decreased sweating	.89	.15	.98	.22	0.39
Hoarseness	.79	.27	.98	.21	0.13
Paresthesias	.91	.22	.98	.21	0.18
Dry skin	.95	.23	.99	.20	0.44
Constipation	.95	.21	.98	.22	0.63
Impaired hearing	.72	.11	.98	.21	0.04
Weight gain	.96	.24	.98	.20	0.67

Correlation of questionnaire scores with T3 values

The correlation of T3 with each of the questionnaire scores was done using Spearman's correlation for nonparametric variables. T3 values among euthyroid patients showed a significant negative correlation with each of the individual questionnaires: TSQ, GHQ-12, HADS-A, HADS-D, and ESS (Table 3). BMI had a negative correlation with T3, which was not significant (P=0.761).

**Table 3:** Correlation of questionnaire scores and BMI with T3 values P-value determined by Spearman correlation method. T3, triiodothyronine; GHQ, General Health Questionnaire; TSQ, thyroid symptom questionnaire; HADS, Hospital Anxiety and Depression Scale; ESS, Epworth Sleepiness Score.

Variable	Correlation (r)	P value
BMI	-0.030	0.761
TSQ	-0.331	0.001
GHQ-12	-0.292	0.002
HADS-A	-0.318	0.001
HADS-D	-0.233	0.015
ESS	-0.205	0.034

The correlation was again reassessed for all questionnaires for T3 values in the lower half of the median for T3 (0.93 ng/ml) from the cohort. Here, the correlation showed statistical significance for TSQ (P=0.007) and GHQ-12 (P=0.013) (Table 4).

**Table 4:** Spearman’s correlation with median T3 <0.93 ng/ml P-value calculated by Spearman correlation. T3, triiodothyronine.

Variable	Correlation (r)	P value
BMI	-0.217	0.127
TSQ	-0.372	0.007
GHQ-12	-0.344	0.013
HADS-A	-0.075	0.599
HADS-D	-0.101	0.480
ESS	0.008	0.956

## Discussion

Among the 108 subjects (98 women and 10 men, age range 18-71 years), the baseline characteristics (Table 1) were as follows: a mean TSH, T4, and T3 of 2.38±1.15 mIU/mL, 9.6±1.89 mcg/dL and 0.97±0.21 ng/mL, respectively. The mean BMI of the cohort was 26.83±4.93 kg/m^2^, which was above the Asian ‘overweight’ cutoff of 23 kg/m^2^.

Among the subjects, residual symptoms were common, affecting most of the biochemically euthyroid subjects, with 82 individuals (75.9%) reporting at least one symptom. This was significantly more than what was reported by Hidalgo et al. [14] (28%) and by Saravanan et al. [15] (46.8%). The impact of T3 levels in biochemically euthyroid patients based on the presence and absence of said symptoms were not statistically significant between the groups except for impaired hearing (P=0.04), which was noted in 2.8% of the subjects. Hearing impairment and audiological alterations assessed by multiple parameters were significantly higher in adults with euthyroid hypothyroidism in a study by Topaloğlu and Şahin [19]. Overall, the higher percentage of residual symptoms among the ethnic Indian population seems to be higher, as corroborated by a similar study from India [17].

The mean score on the thyroid symptom questionnaire, TSQ (scored 0-3 ordinally for ‘not at all’, ‘no more than usual’, ‘rather more than usual’ and ‘much more than usual’ response categories), which was chosen as a means to assess how patients felt on LT4 therapy, was 9.99±6.48 (range: 0-28 on a maximum score of 36). In the study by Saravanan et al. [15] in the United Kingdom that assessed 597 hypothyroid subjects and 551 euthyroid controls, TSQ scores varied significantly among groups; with 35% of controls, 46.8% of patients and 8.6% of euthyroid patients scoring ≥3 on the TSQ (P<0.001 for patients vs. controls, and for euthyroid patients vs. controls). Though the manner of scoring each of the subject response categories and thus the cutoffs vary widely among studies, our data shows the patients on average have higher TSQ scores on LT4 supplementation, suggesting a greater level of treatment dissatisfaction. Furthermore, the TSQ score showed a strong inverse correlation with T3 values (coefficient r=-0.331, P=0.001). The above findings suggest that TSQ is a useful tool for screening not only for symptoms of hypothyroidism in general, but also a tool to identify patients with residual symptoms on LT4 supplementation.

The 12-item General Health Questionnaire (GHQ-12) was chosen as a mental health and morbidity screening tool. On application of GHQ-12 in this cohort of patients (which excluded patients with a history of psychiatric illness or who are on antipsychotic or antidepressant medication), it showed a mean score (scored 0-3 for ‘better than usual’, ‘same as usual’, ‘less than usual’ and ‘much less than usual’ response categories) of 9.83±5.15 (range: 0-33 with a maximum score of 36). When scored similarly as in this study, a cutoff of ≥10 was used to indicate possible mental distress [18, 19] with higher scores showing more severe distress. As many as 57 of 108 (52.7%) subjects in the study had met this cutoff. Furthermore, there was a significant negative correlation between T3 values and GHQ-12 scores (r=-0.292, P=0.002). In a community study from south India by John et al., 16% of the population satisfied international criteria for common mental disorders [20]. Though GHQ-12 is not validated for use in patients with hypothyroidism or for assessing euthyroid patients, the data suggests a higher prevalence of ‘mental distress’ among euthyroid patients in the current study.

We used the HADS tool for assessing anxiety and depression, a brief 14-item self-administered tool for reporting anxiety and depression (HADS-A and HADS-D). Both anxiety and depression are connected to overt hypothyroidism and their impact after biochemical euthyroidism is achieved with LT4 is not well known. Though anxiety is more linked with hyperthyroidism, it is also well documented among hypothyroid patients [21, 22]. The HADS-A and HADS-D scores for anxiety and depression, respectively, were 4.39±4.02 and 3.09±3.03 (range: 0-16 and 0-12, respectively; maximum score of 21 each). Both HADS-A and HADS-B scores had a significant negative correlation with T3 in euthyroid patients in the present study, especially HADS-A (r=-0.318, P=0.001 for HADS-A and r=-0.233, P=0.015 for HADS-B). As with GHQ-12, the HADS tool is not validated for use among euthyroid patients in the current context though scores seem to suggest worse outcomes among this population [23].

Following the assessment for obstructive sleep apnea by ESS, the mean score was 3.76±2.66 (range:0-12, maximum score of 24). Even though scores showed a significant negative correlation with T3 scores (r=-0.205, P= 0.034) in this study, the confounding effect of a higher mean BMI in our sample helps to draw no meaningful conclusion even though studies show its association in hypothyroid patients especially among males [24,25].

In the analysis for correlation between T3 below the median for the cohort (<0.93 ng/ml), a significant correlation was noted for TSQ (P=0.007) and GHQ-12 (P=0.013), which reinforces its utility in patients with lower T3 values compared to other questionnaires for better identifying patients with residual symptoms.

We acknowledge the lack of controls in the present study is a drawback, which would have helped diagnose unintended factors and better aid in the measurement of errors.

## Conclusions

Treatment satisfaction remains suboptimal for patients with residual symptoms of hypothyroidism who are biochemically euthyroid. The frequency and severity of residual symptoms reported in the current study appear to be more prevalent than those reported elsewhere. TSQ especially is a useful tool to recognize the residual symptoms in addition to its use to assess well-being among patients treated with LT4. This utility of TSQ opens further possibilities with intervention in such patients by oral T3 supplementation. Such treatment may help alleviate other affected domains like mental health, anxiety, and depression as shown here by other questionnaire tools.
